# Inactivation and Subsequent Growth Kinetics of *Listeria monocytogenes* After Various Mild Bactericidal Treatments

**DOI:** 10.3389/fmicb.2021.646735

**Published:** 2021-03-19

**Authors:** Taisong Fang, Yufan Wu, Yani Xie, Linjun Sun, Xiaojie Qin, Yangtai Liu, Hongmei Li, Qingli Dong, Xiang Wang

**Affiliations:** ^1^School of Medical Instrument and Food Engineering, University of Shanghai for Science and Technology, Shanghai, China; ^2^Research Centre of Analysis and Test, School of Chemistry and Molecular Engineering, East China University of Science and Technology, Shanghai, China

**Keywords:** *Listeria monocytogenes*, inactivation, growth parameters, sublethal injury, pathogens control

## Abstract

This study was carried out to investigate the effects of mild heat, lactic acid, benzalkonium chloride and nisin treatments on the inactivation, sublethal injury, and subsequent growth of *Listeria monocytogenes*. Results showed that the Bigelow model successfully described the thermal inactivation kinetics, while the Log-linear model with tail consistently offered the most accurate fit to LA, BC, and nisin inactivation curves of cells. Differential plating indicated that percentage of sublethal injury for nisin treated cells was significantly higher than that for the other three treatments. Compared to non-treated cells, significant extension of lag time was observed for all treated cells. The longer exposures to heat treatment contributed to the extended lag time of the survivors. While for LA, BC and nisin treated cells, the longest lag time was not observed at the most severe treatment conditions. The correlation analysis of sublethal injury percentage on the duration of lag time revealed that only heat treatment showed the significant correlation. Overall, the lag time analysis could evaluate a wide range of bacterial injury. Lag time of treated cells was significantly influenced by stress treatments and temperatures of recovery, however, there were not any significant changes in the maximum specific growth rate between treated and non-treated cells under isothermal recovery conditions. The information generated from this study is valuable for utilizing intervention strategies in the elimination or growth inhibition of *L. monocytogenes*.

## Introduction

*Listeria monocytogenes* is the causative agent of human listeriosis, a life threatening foodborne disease commonly associated with consumption of contaminated food products, especially ready-to-eat (RTE) foods (Swaminathan and Gerner-Smidt, 2007). Previous studies reported that fatality rate of listeriosis was up to 15.6% in European Union in 2018 (European Centre for Disease Prevention and Control [ECDC], 2019). In China, in spite of rare listeriosis outbreaks, high contamination rate was reported in retail foods, which could also cause a high potential risk to human health (Wu et al., 2016). Due to high fatality rate of listeriosis and high tolerance of *L. monocytogenes* against food processing stresses such as low temperature, pH, a_w_ or high salinity, the bacterium has been identified as the one of the most dangerous pathogens associated with food products (Ferrentino et al., 2015). Therefore, it is vital to understand the response of *L. monocytogenes* to multiple food related stresses, and take into account growth kinetic parameters of survivors in different environments in order to design appropriate intervention strategies to control the level of *L. monocytogenes* in food chain.

Two important parameters inherent to *L. monocytogenes* growth kinetics are the lag time (λ) and the maximum specific growth rate (μ*_*max*_*), and it is necessary to predict the two parameters accurately because outgrowth of *L. monocytogenes* is unacceptable in food products. The lag time reflects an adjustment period during which bacterial cells repair injuries caused by any stress, and modify themselves in order to initiate exponential growth in the new environment. Compared to reliable information on the maximum specific growth rate, the lag time is usually difficult to be predicted accurately due to poor understanding of initial physiological state of cells and/or repair of injured cell structures (D’Arrigo et al., 2006). The duration of lag time depends on numerous factors, including actual growth environments such as physical or chemical conditions. In addition, the history (physiological state in previous environments) of cells can also significantly influence the lag time in the actual growth environments, many studies have demonstrated that bacterial cells show shorter lag time when changes are smaller between previous and actual growth conditions (Francois et al., 2007; Yue et al., 2019). However, *L. monocytogenes* is routinely exposed to bactericidal treatments such as heat, organic acids, quaternary ammonium compounds and bacteriocins stresses (Shi et al., 2013; Humayoun et al., 2018) in food or food processing environments, and mild bactericidal treatments for food preservation are being utilized to obtain microbiologically safe food products and satisfy consumers’ demands for minimally processed foods. The mild process may result in surviving *L. monocytogenes* cell populations, which most likely exhibited the state of sublethal injury. This suboptimal physiological state of cells could considerably extend the duration of lag time because of self-repairing process of injured cells in the appropriate growth environments (Yuste et al., 2004). After resuscitation, *L. monocytogenes* possesses full virulence, giving rise to a threat to public health. Therefore, injury induced by exposure to the mild bactericidal treatments influences the growth behavior of surviving cells. This emphasizes the importance of understanding the growth of *L. monocytogenes* after different mild bactericidal treatments.

In the present study, we investigated the effects of three types of treatments (physical, chemical, and biological bactericidal treatments) with heat, lactic acid (LA), benzalkonium chloride (BC) and nisin on the inactivation, sublethal injury, and subsequent growth of *L. monocytogenes*. Stress exposure conditions were selected to encompass various potential sublethal stresses encountered by *L. monocytogenes* in foods or food processing environments. In addition, the effects of various recovery temperatures (20, 25, 30, and 37°C) on growth parameters of treated *L. monocytogenes* were also determined.

## Materials and Methods

### Bacterial Strain and Culture Conditions

*Listeria monocytogenes* (ATCC 19112) purchased from the China Center of Industrial Culture Collection (Beijing, China) was used in this study. Frozen stocks of bacteria were maintained in Tryptone Soy Yeast Extract Broth (TSB-YE; Beijing Land Bridge Technology Co., Ltd., Beijing, China) with 50% glycerol at −80°C. Working stocks were stored at 4°C on Tryptone Soy Agar with 0.6% Yeast Extract (TSA-YE; Beijing Land Bridge Technology Co., Ltd., Beijing, China) and were renewed monthly. Prior to each experiment, a single colony was inoculated into 10 mL of TSB-YE and incubated in a shaker with 110 rpm at 37°C for 16–18 h. Then 100 μL of overnight culture was transferred into 10 mL of fresh TSB-YE and incubated at same conditions to yield stationary phase cells which contained approximately 10^9^ CFU/mL cells.

### Mild Bactericidal Treatments

Four mild bactericidal treatments included one physical treatment with heat, two chemical treatments with LA and BC, and one biological treatment with nisin. Before each treatment experiment, 1 mL stationary phase cultures were centrifuged at 5,000 *g* for 10 min (Thermo Fisher Scientific Co., Ltd., Shanghai, China). Harvested cells were washed twice with 0.85% saline solution and re-suspended in 1 mL 0.85% saline solution to yield a cell density of ca. 10^9^ CFU/mL. Stress exposure conditions were adjusted to suitable parameters leading to approximately 1.5–2.5 log CFU/mL reduction in cell counts. After each stress exposure, the surviving cells were placed at 25°C for recovery and subsequent growth. The lag time was monitored by using TTD (Time to Detection) method based on optical measurements (see below).

#### Heat Treatment

Mild heat treatment of *L. monocytogenes* was performed at 48°C for different treatment time (30, 60, 90, 120, and 150 min) according to preliminary experiments. It was carried out using thin walled PCR tubes (Shanghai Generay Biotech Co., Ltd., Shanghai, China) containing 30 μL culture and a thermal cycler instrument (Analytik Jena AG, Germany). The heating program was initially set at 37°C for 1 min in order to reduce and standardize the time to reach the target heat treatment temperature, and test tubes were removed at the set time intervals after the cultures had reached 48°C (Wang et al., 2017). After heat treatment, PCR tubes were immersed immediately in an ice-water bath for 1 min. Subsequently, decimal dilutions were made and plated, and then incubated at 37°C for 48 h before surviving cells’ enumeration.

#### Lactic Acid, Benzalkonium Chloride, and Nisin Treatments

Stress treatments were performed by incubating *L. monocytogenes* cells with corresponding solutions at 25°C in a static incubator (Keer Equipment Co., Ltd., Nanjing, China). LA (40 mmol/L, Kuling Fine Chemical Co., Ltd., Shanghai, China), BC (70 mg/L, Macklin Biochemical Co., Ltd., Shanghai, China) and nisin solution (900 IU/mL, Meryer Chemical Technology Co., Ltd., Shanghai, China) was separately prepared by dissolving the solute in 0.85% saline, and sterilized by filtration through 0.22 μm membrane filter units. For LA stress exposure, 1 mL harvested stationary cells were re-suspended in 1 mL LA solution for different treatment time (20, 40, 60, 80, and 100 min). For BC treatment, *L. monocytogenes* cells were incubated for 12, 24, 36, 48, 60, and 72 min, respectively. For nisin, cells were treated from 12 to 60 min and removed at a time interval of 12 min. Treated cells were harvested by centrifugation at 5,000 *g* for 10 min, and re-suspended in 1 mL TSB-YE for further analysis.

#### Viable and Sublethally Injured Cell Counts

The counts of *L. monocytogenes* were enumerated by a traditional plating method. Each sample was serially (1:10) diluted with 0.85% NaCl solution and appropriate dilutions were plated on TSA-YE (the non-selective medium) and TSA-YE with 5% NaCl (the selective medium) (Uyttendaele et al., 2008). Both vital and injured cells were able to grow on TSA-YE, while those which appeared on TSA-YE with 5% NaCl were regarded as only uninjured cells (Ray et al., 1978). Following formula (Busch and Donnelly, 1992) was used to calculate the percentage of sublethally injured cells:

(1) $$\text{The injury rate}\left(\%\right)=\left(1-\frac{\mathrm{counts}\,\mathrm{on}\,\mathrm{selective}\,\mathrm{medium}}{\mathrm{counts}\,\mathrm{on}\,\mathrm{non}-\mathrm{selective}\,\mathrm{medium}}\right)\times 100\%$$

Time-averaged injured cells coefficient (TICC) was calculated to quantify the sublethally injured cells for the whole treatment time, and the equation is as follows (Miller et al., 2006):

(2) $$T\,I\,C\,C=\frac{\int_{t_{i\,\text{nitial}}}^{t_{\text{final}}}\left[\%\text{Sublet}\mathrm{hal}\mathrm{Injury}\left(t\right)\right]dt}{t_{\text{final}}-t_{\text{initial}}}$$

Where *t* is the bactericidal treatment time, *t*_*initial*_ and *t*_*final*_ are the first and last sampling time, respectively.

### Optical Density Measurements

The effects of heat, LA, BC, and nisin treatments on the subsequent growth of *L. monocytogenes* were determined by growth curves using an automatic Bioscreen C system (Oy Growth Curves Ab Ltd., Helsinki, Finland). Treated and non-treated cultures were serially diluted (1:10) in TSB-YE, and 200 μL volume of different dilutions with concentrations ranged from 10^6^ to 10^2^ CFU/mL were added to 200 wells of two honeycomb plates. The honeycomb plates were placed in the Bioscreen C at an incubation temperature of 25°C, and the growth of *L. monocytogenes* was monitored by reading OD_600_ of the wells at 10 min intervals. For each well, the time to reach an OD_600_ of 0.15 from the start of incubation (OD_600_ = 0.10) was determined, and a cell concentration of approximately 10^7^ CFU/mL corresponding to an OD_600_ value of 0.15 was determined by the count of plated viable cells. Honeycomb plates were shaken at medium intensity for 20 s before every measurement, and each stress experiment was repeated three times. After heat treatment for 150 min, LA treatment for 20 min, BC treatment for 48 min and nisin treatment for 24 min, treated cells were incubated at 20, 25, 30, and 37°C to compare the effects of recovery temperatures on growth parameters of treated *L. monocytogenes* by using TTD method described above.

### Estimation of Growth Parameters of *Listeria monocytogenes*

The growth parameters of treated and non-treated *L. monocytogenes* were calculated based on TTD method. The μ*_*max*_* value was calculated as the reciprocal of the absolute value of the regression slope of *T*_*d*_ (detection time) versus natural logarithm of initial cell concentration. The initial cell concentration for each dilution was determined by serial dilution and plating on TSA-YE, followed by incubation at 37°C for 48 h. Lag time of *L. monocytogenes* was estimated based on the following equation (Baranyi and Pin, 1999):

(3) $$\lambda =T_{d}-\left[\frac{l\,n\,\left(N_{d}\right)-l\,n\,\left(N_{0}\right)}{\mu_{m\,a\,x}}\right]$$

Where, *N*_*d*_ is the bacterial number at the turbidity detection level (CFU/mL); *N*_0_ is the initial concentration of cells (CFU/mL); *T*_*d*_ is the turbidity detection time (h); μ*_*max*_* is the maximum specific growth rate (h^–1^) under the experimental conditions described above.

### Modeling Inactivation Kinetics of *Listeria monocytogenes*

After each treatment time, the counts of *L. monocytogenes* were converted to log_10_ values, and the survivors (log_10_ *N*_*t*_) were represented vs. the treatment time (min for heat, LA, BC and nisin treatments) to construct survival curves. The Bigelow model and Log-linear model with tail were, respectively, used to fit the linear and non-linear inactivation kinetics obtained in our selected conditions, and inactivation parameters were obtained on the software GInaFiT (version 1.6) (Geeraerd et al., 2005). The goodness of fitting was evaluated by root mean squared error (RMSE). The equations and relevant parameters of the selected models are as follows (Bigelow, 1921; Geeraerd et al., 2000):

(4) $$L\,o\,g_{10}\,\left(N_{t}\right)=L\,o\,g_{10}\,\left(N_{0}\right)-k_{m\,a\,x}\,t/L\,n\,\left(10\right)$$

(5) $$Log_{10}\left(N_{t}\right)=Log_{10}\{\left[10^{L\,o\,g_{10}\,\left(N_{0}\right)}-10^{L\,o\,g_{10}\,\left(N_{r\,e\,s}\right)}\right]\times e^{-k_{m\,a\,x}\,t}+10^{L\,o\,g_{10}\,\left(N_{r\,e\,s}\right)}\}$$

Where, *N*_*t*_ represents the counts of survivors (CFU/mL); *N*_0_ represents the initial counts (CFU/mL); *k*_*max*_ is the specific inactivation rate (min^–1^); *t* is the exposure time of each treatment (min); *N*_*res*_ represents the residual population (CFU/mL).

### Modeling the Effects of Different Temperatures on Growth Parameters

The λ and μ*_*max*_* values of non-treated and treated *L. monocytogenes* cells were further analyzed as a function of recovery temperatures to develop the secondary model. The equations and relevant parameters of the models are as follows (Ratkowsky et al., 1982):

(6) $$\sqrt{\mu_{m\,a\,x}}=b\times \left(T-T_{0}\right)$$

(7) $$\frac{1}{\sqrt{\lambda }}=b\times \left(T-T_{0}\right)$$

Where, λ is the lag time (h); μ*_*max*_* is the maximum specific growth rate (h^–1^); *b* is the regression constant; *T*_0_ is the minimum temperature required for growth of *L. monocytogenes* (°C); *T* is the recovery temperature (°C).

### Statistical Analysis

All experiments were repeated three times, means and standard deviations were determined from independent triplicate trials. Single-factor analysis of variance (ANOVA) and Tukey’s test by SPSS 25 (SPSS Inc., Chicago, IL, United States) was used to test for any significant difference between means for sake of multiple comparisons. Correlation coefficients were calculated to conduct the correlation analysis of stresses for λ value. A significant level of 0.05 with *p* value was used in each case.

## Results and Discussion

### Inactivation of *Listeria monocytogenes* by Mild Bactericidal Treatments

**FIGURE 1:**
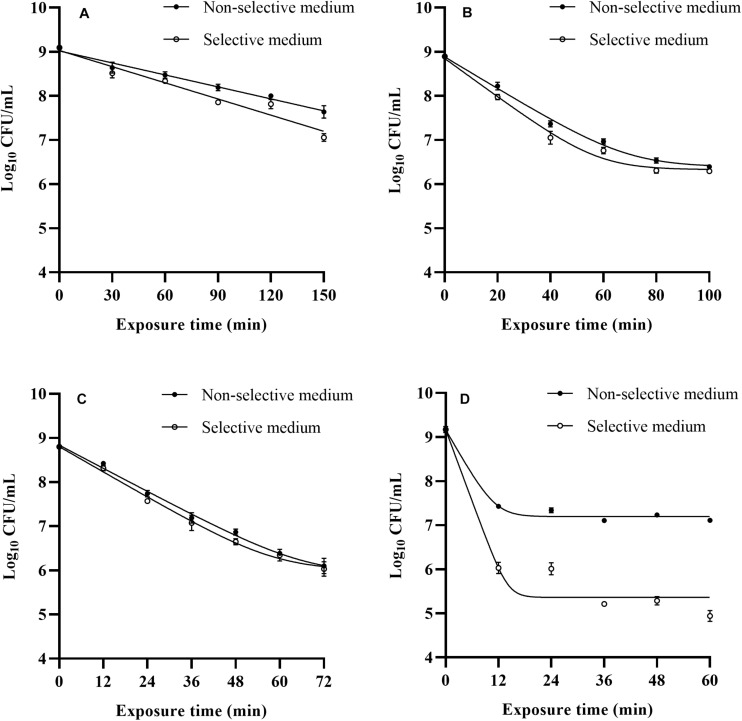
Observed survival curves of *L. monocytogenes* after 48°C **(A)**, LA **(B)**, BC **(C)**, and nisin **(D)** treatments.

The survival of *L. monocytogenes* subjected to the four bactericidal treatments was evaluated, and the survival curves obtained from selective and non-selective media are shown in Figure 1. Overall, the inactivation curves exhibited two different patterns, log-linear and log-linear with tail. The Bigelow model successfully described the inactivation curves of heat treated cells. Similar thermal inactivation kinetics were also observed by Wang et al. (2017) and Haskaraca et al. (2019) while performing mild heat treatments of *L. monocytogenes*. The Bigelow model and relevant D value parameter have been extensively used to describe the microbial thermal inactivation in many studies. However, more and more reports have emphasized that non-linear models are more suitable than log-linear model for describing microbial heat inactivation curves, especially for mild heat treatments (Augustin et al., 2010; Aryani et al., 2015). A study of Marcén et al. (2019) indicated that mild heat (58°C) inactivation curve showed a shoulder. Similar results were also found in the thermal inactivation kinetics of *Salmonella enterica* (Wang et al., 2017). The Shoulder phenomena can be attributed to the presence of sublethally injured cells in the treated population, and their subsequent accumulation leading to sublethal injury during the manifestation of shoulders (Lou and Yousef, 1997). In fact, many studies have supported that heat inactivation kinetics present shoulders or tails, which are often fitted with the Weibull model (Aryani et al., 2015; Arioli et al., 2019). In this context, when estimating the effectiveness of heat treatments, non-linear kinetics could be taken into account upon existence of shoulders or tails.

The log-linear model with tail consistently offered the most accurate fit to LA, BC, and nisin inactivation curves of cells recovered from both media based on the small RMSE values (RMSE ≤ 0.133, data not shown). Many authors focused their attention on inactivation level of foodborne pathogens after exposure to a fixed LA or BC treatment time (Shi et al., 2017; Kang et al., 2019; Andrade et al., 2020), but there are limited studies on relevant inactivation kinetics of *L. monocytogenes*. In this study, tails appeared at an inactivation level of approximate 2 log cycles, especially for nisin treatment. When 900 IU/mL nisin was applied, the *L. monocytogenes* counts reduced significantly (*p* < 0.05) as the treatment time increased from 0 to 12 min, and counts of survival cells remained approximate 7 log CFU/mL in 12–60 min stress exposure. The inactivation kinetics of *L. monocytogenes* cells to nisin treatment obtained in the present study were in agreement with that of Shi’s study (Shi et al., 2013). The occurrence of tails could be attributed to different factors, such as the presence of resistant cells subpopulation, adaptation phenomena along the treatment time.

Results in our study reflected differences among inactivation curves depending on the treatments applied, especially the inactivation kinetics of *L. monocytogenes* cells exposure to three treatments showing tail phenomena, which indicated possible adaptation phenomena along the treatment time. Therefore, from a practical point of view, when mild bactericidal treatments are designed, determination of inactivation kinetics should be taken into account to select process conditions applicable and avoid overestimation of bactericidal effectiveness, and further studies can be conducted by a deeper knowledge of their mode of action on foodborne pathogens to obtain a better profit of all these bactericidal technologies.

### Stresses Induced Sublethal Injury Based on Differential Plating

**FIGURE 2:**
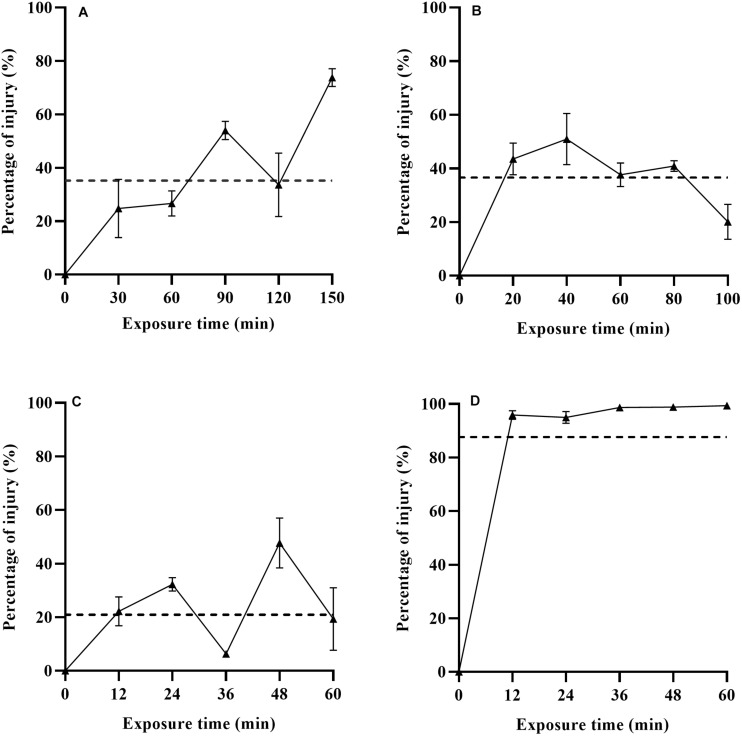
Percentage of sublethal injured *L. monocytogenes* cells and TICC values (dashed line) after exposure to heat **(A)**, LA **(B)**, BC **(C)**, and nisin **(D)** stresses based on differential plating.

The percentage of *L. monocytogenes* injury based on differential plating and TICC values after exposure to heat, LA, BC and nisin stresses are shown in Figure 2. The percentage of injury to heat, LA, BC, and nisin stresses ranged from 24.80–73.79, 20.05–50.93, 6.35–47.70, and 94.98–98.85%, respectively, which indicated the existing of different level of injured cells. Percentage of injury for non-treated cells was equal to zero (data not shown). In case of nisin treatment, the estimated ratio of injured bacterial cells was over 95% after 12 min exposure, and it was close to 100% as the treatment time went on (Figure 2D). As a whole, the percentage of injury in nisin treated cells was significantly higher than that in the other three treatments (*p* < 0.05), and this is also confirmed by the TICC value for the whole nisin treatment time (87.62%). Nisin has effective antimicrobial activities against Gram-positive bacteria including *L. monocytogenes*, and the cytoplasmic membrane is the target for nisin action (Martins et al., 2010). The TICC value in the present study revealed that nisin stress caused the high degree of cell membrane damage, which was also an indication of mechanism of relevant antimicrobial action. The possible reason for high sublethal damage rather than direct inactivation is that adaption phenomena along the treatment time induced the emergence of nisin-resistant cells subpopulation. This is demonstrated by Harris et al. (1991), which has described the presence of nisin-resistant mutants after exposure of nisin-sensitive *L. monocytogenes* cells to relatively high concentrations of nisin. Resistance could be associated with a barrier including changes in fatty acid and phospholipid composition of the cytoplasmic membrane which prevent the nisin from crossing the barrier (Davies et al., 1996). Actually in food matrices, nisin has low solubility and can interact with phospholipids and proteins, which lead the efficacy of nisin to last for only a short time (Chen et al., 2014). From a practical point of view, combination of other bactericidal technologies and nisin could overcome the limitation of nisin. Moreover, the application of nisin at lower concentrations could also reduce the rate of emergence of nisin-resistant cells along the treatment time.

When subjected to the other three stresses (heat, LA, and BC), *L. monocytogenes* had low percentage of injury, especially for BC treatment. For heat treatment, the evaluated proportion of heat injured cells had an increasing trend with increasing treatment time (0–150 min). Kawasaki et al. (2018) have reported similar results for *Salmonella* Enteritidis in PBS and ground beef at 52.5°C for 0–60 min. As is shown in Figure 2, the TICC values of heat, LA and BC induced injured *L. monocytogenes* cells were only 35.20, 36.60, and 20.89%, respectively. It has been described before that the LA treatment (1,000 mmol/L, 4 min) resulted in almost 100% sublethal injury of *E. coli* O157:H7 (Smigic et al., 2009). This could be associated with the different bacteria, different concentrations and exposure time of LA used in their studies. The use of LA has been an authorized decontaminating treatment in beef production, which was proposed by the European Food Safety Authority in 2011 (EFSA Panel on Biological Hazards [BIOHAZ], 2011). In addition, BC, as a kind of Quaternary Ammonium Compounds (QACs), has been widely used for disinfecting the surfaces in food production environments (To et al., 2002). In food industry, bacteria are inevitably exposed to sublethal concentrations of sanitizing compounds, and this could induce injured cells subpopulation or adaption of initially susceptible bacteria. Therefore, evaluation of the degree of sublethal injury is critical to the safety of final products that have undergone food processing.

### Growth Lag Time of *Listeria monocytogenes* After Mild Bactericidal Treatments

**FIGURE 3:**
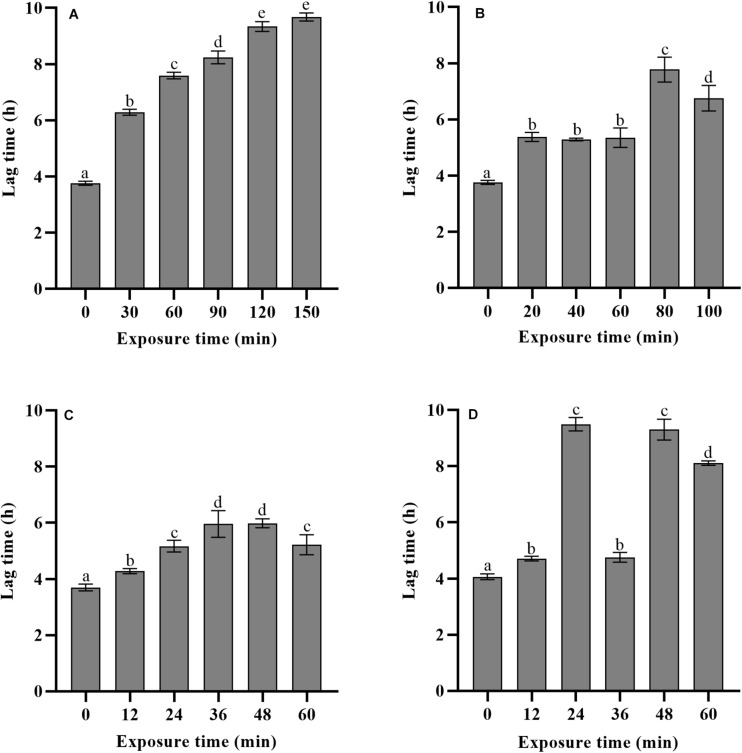
Effects of different exposure time of heat **(A)**, LA **(B)**, BC **(C)**, and nisin **(D)** stresses on the lag time of *L. monocytogenes.*

**FIGURE 4:**
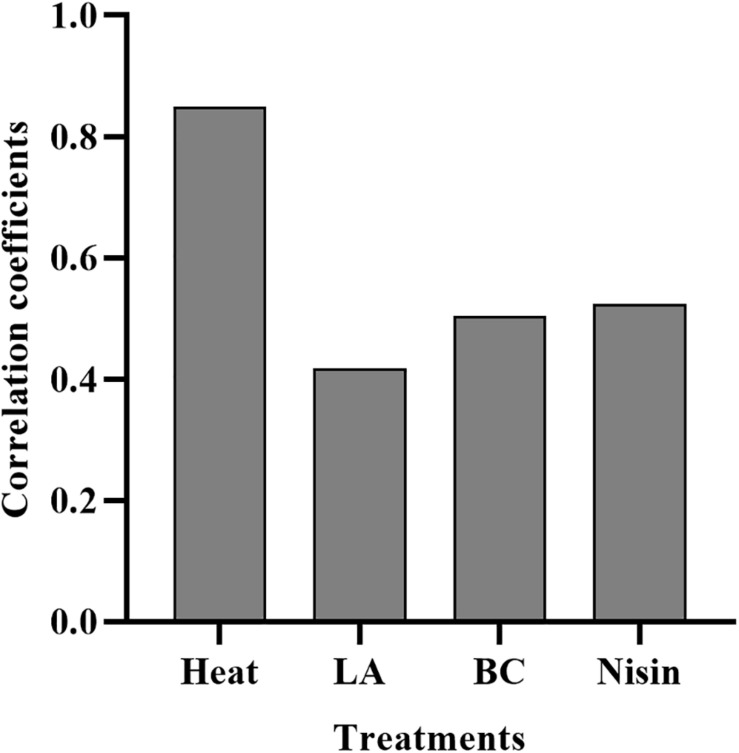
Correlation coefficients of heat, LA, BC, and nisin treatments induced percentage of injury for lag time.

The lag time prediction of foodborne pathogens is useful for microbial risk assessment. To accurately predict and then control the growth of *L. monocytogenes* in food products, it is important to understand the effects of various stresses experienced history on the lag time. In the present study, TTD method based on the Baranyi growth model was used to monitor *L. monocytogenes* growth after mild bactericidal treatments. This method is known for its high efficacy to estimate the λ and μ*_*max*_* values without requirement of calibration between cell numbers and absorbance. The effects of heat, LA, BC, and nisin stresses on the lag time of *L. monocytogenes* are shown in Figure 3. Compared to non-treated cells, observed significant extension of lag time was a direct consequence of prior sublethal injury (*p* < 0.05). When the final reductions in the number of viable cells were 1.5–2.5 log units, lag time was significantly different among the four treatments with heat treated cells exhibiting the longest lag time, followed by that of nisin, LA and finally BC treated cells. Heat treated cells showed the lag time in the range of 5.86–9.68 h. The longer time the heat treatment was the longer was the lag time of survivors. In contrast, for LA, BC, and nisin treated cells, longer exposure time did not reveal the longer lag time. Moreover, it did not correspond to the maximum level of injury obtained by differential plating. To compare the effects of sublethal injury percentage on lag time, the correlation coefficients have been evaluated and illustrated in Figure 4. The correlation coefficient of heat treatment for lag time was highest (0.850), and value close to 1 indicated almost linear positive correlations (*p* < 0.05). Furthermore, the correlation coefficients of LA, BC and nisin treatments were 0.42, 0.51, and 0.52, respectively. However, the results of statistical analysis revealed no significant correlations between percentage of injury and lag time of LA, BC, and nisin treated cells (*p* > 0.05). As a whole, these results would indicate that only heat induced injury and lag time of *L. monocytogenes* exhibited a significant correlation.

Four mild bactericidal treatments used in the present study are some of the most studied decontamination treatments with heat, organic acids, quaternary ammonium compounds and peptide antimicrobial agents. As expected, compared to non-treated cells, significant extension of lag time revealed the existence of the injured cells in the surviving population for all conditions tested, which was consistent with the results of other studies. For instance, in the study of Xuan et al. (2017), the effect of injury caused by heat (55°C) on the growth parameters indicated that larger λ value was observed in heat injured *L. monocytogenes* as compared to control group. Similar results were also found in the λ value of injured *L. monocytogenes* cells after acid (pH 4.2), osmotic (10% NaCl) and heat (55°C) stresses (Sibanda and Buys, 2017). Up to now, many authors have focused their attention on the lag time of injured foodborne pathogens after exposure to a fixed bactericidal treatment time, however, not much literature is available on how the lag time changes after different treatment time.

Sublethal injury of foodborne pathogens has huge implications on food safety, and ability to detect injured microorganisms is critical since injured cells can resuscitate and then possess full virulence in a favorable environment. To estimate the amount of sublethally injured cells after bactericidal treatments, conventional selective techniques and other detection methods such as flow cytometry and fluorescent metabolic probes have been used in most studies (Uyttendaele et al., 2008; Zhang et al., 2020). According to these methods, the proportion of injured cells in the population can be calculated, with the outcome largely dictated by incubation conditions (such as temperatures and atmospheres) and detection methods. Sibanda and Buys (2017) assessed the degree of stress induced injury by using differential plating and flow cytometry coupled with membrane integrity indicators. The results indicated that both methods showed significant difference among stress treatments, and membrane integrity was not a sufficient indicator of heat stress injury. For differential plating method, due to the differences in recovery ability of bacteria, the percentages of injury also depend on the types of selective media. For instance, selective media based on NaCl supplement can only detect the sublethal membrane damage of cells, while several other damaged cellular targets such as enzymes, RNA and DNA can’t be detected, so this outcome implies a potential for under-estimation of injury (Miller et al., 2006). In addition, calculated percentage of injury based on differential plating method only indicates the portions of amounts of injured cells in the population (it may range from 0 to 100%), which reflect all cells in the population are at the same degree of injury. However, due to individual cell heterogeneity, responses to stress exposures differ among stress sensitive and resistant cell subpopulations, so injured populations are often a mixture of cells in different physiological states (various degree of injury) (Casadesús and Low, 2013), some cells in the population are more damaged than others. Therefore, although differential plating method can evaluate percentage of amounts of injured cells, this method only reflect the level of injury (based on the amounts of injured cells) rather than the degree of injury (based on different physiological states).

It is well known that compared to uninjured cells, injured cells can present the extension of lag time due to the resuscitation behavior, so the lag time length of injured cells might be useful for evaluating the degree of sublethal injury. Kawasaki et al. (2018) described the growth delay time (GDT, the difference of λ value between treated and non-treated cells) of *Salmonella* Enteritidis by real-time PCR monitoring assay, which was then compared with traditional culture method. Results showed that calculated percentage of injured cells was 100% during 24–48 min treatment since bacterial colonies were not detected on selective medium. The differential plating method was only able to evaluate the degree of injury until a stress exposure time of 12 min. However, the GDT significantly increased with the prolonged treatment time (24–48 min), which indicated that GDT could evaluate a wider range of bacterial injury than the traditional culture method. In the present study, as are shown in Figures 2, 3, a similar finding was also observed in case of nisin treatment, the estimated ratio of injured bacterial cells was 98.71% after 36 min exposure, which was not significantly different among different treatment time (36–60 min), thus indicating constant level of sublethal injury along the treatment time by traditional culture method. However, the λ value obtained by TTD method revealed that injury to microbial cells was still in a dynamic change. The present findings revealed the significance of lag time analysis to evaluate a wide range of bacterial injury, which can quantify the degree of injury more accurately than differential plating method.

### Growth Parameters Under Different Recovery Temperatures

The λ and μ*_*max*_* values of *L. monocytogenes* after heat, LA, BC, and nisin treatments at recovery temperatures of 20, 25, 30, and 37°C were evaluated (Table 1). For all the treatments, significant shorter λ and higher μ*_*max*_* values were observed at higher recovery temperatures. Stress treatments and recovery temperatures had significant effects on λ values (*p* < 0.05), while regardless of stress treatments, μ*_*max*_* only varied significantly at recovery temperature from 20 to 37°C (*p* < 0.05). When compared to non-treated cells, λ values for treated cells were significantly higher (*p* < 0.05) at each recovery temperature, and there were no great change in the μ*_*max*_* values between treated and non-treated cells under isothermal recovery conditions in spite of results of statistical analysis indicating significant difference in some cases.

**TABLE 1:** Growth parameters of *L. monocytogenes* at 20, 25, 30, and 37°C after heat, LA, BC, and nisin treatments.

Treatment conditions	Recovery temperature (°C)	λ (h)	μ*_*max*_* (h^–1^)
Control	20	5.34 ± 0.20 Aa	0.430 ± 0.009Aa
	25	3.72 ± 0.09 Ba	0.626 ± 0.019Ba
	30	3.41 ± 0.10Ca	0.850 ± 0.011Ca
	37	2.45 ± 0.03Da	0.963 ± 0.003Da
Heat	20	11.04 ± 0.16Ab	0.387 ± 0.007Ab
	25	9.68 ± 0.15Bb	0.641 ± 0.007Ba
	30	7.82 ± 0.21Cb	0.837 ± 0.039Ca
	37	4.39 ± 0.11Db	0.956 ± 0.010Da
LA	20	8.49 ± 0.09Ab	0.433 ± 0.011Aa
	25	5.38 ± 0.16Bb	0.632 ± 0.006Ba
	30	4.63 ± 0.10Cb	0.890 ± 0.007Cb
	37	3.94 ± 0.10Db	0.943 ± 0.022Db
BC	20	7.06 ± 0.47Ab	0.428 ± 0.004Aa
	25	5.98 ± 0.16Bb	0.632 ± 0.025Ba
	30	4.37 ± 0.10Cb	0.869 ± 0.007Ca
	37	3.81 ± 0.26Db	0.927 ± 0.024Db
Nisin	20	12.88 ± 0.38Ab	0.435 ± 0.011Aa
	25	9.49 ± 0.24Bb	0.705 ± 0.005Bb
	30	8.06 ± 0.17Cb	0.873 ± 0.012Ca
	37	5.16 ± 0.19Db	0.971 ± 0.010Da

*Values are expressed as means ± standard deviations of three replicate experiments. Means with different uppercase letters in the same column for each treatment condition indicate significant differences (*p* < 0.05). For each recovery temperature, means with different lowercase letters indicate significant differences (*p* < 0.05) between control and each stress treatment.*

**FIGURE 5:**
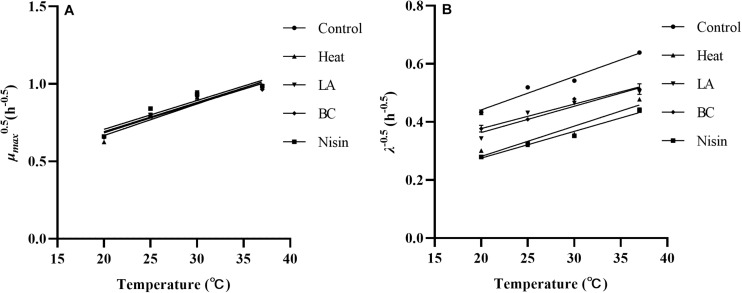
The square root of the μ*_*max*_* **(A)** and reciprocal of square root of the λ **(B)** of *L. monocytogenes* after various treatments as a function of recovery temperatures.

In bacterial growth kinetics, the λ value of cell population is influenced by the physiological state prior to environmental change, especially sublethal injury induced by various stresses (Augustin et al., 2000). From the results of our study, compared to other recovery temperatures, the observed shorter λ values at 37°C for each treatment could be attributed to a quicker repair rate. This could be associated with the relevant protein synthesis, particularly enzymes that contribute to synthesis of membrane lipids necessary for repairing damaged cell membranes (García et al., 2006). In fact, for stress induced injured cells, other functional components such as DNA and RNA of bacterial cells can also be damaged (Chilton et al., 2001), which need to be repaired before cells can commence division again. The μ*_*max*_* of treated *L. monocytogenes* cells showed no significant variation among treatments and was expectedly decreased from 37 to 20°C, which reflected temperature dependence of bacterial growth in the exponential phase. Similar results were also observed in Francois et al.’s (2007), which showed that recovery temperature affected both λ and μ*_*max*_* values, whereas stresses influenced λ value only. To evaluate the effects of recovery temperatures on growth parameters of stresses treated *L. monocytogenes* cells, the square root model was tested. As shown in Figure 5, the reciprocal of square root of λ and the square root of μ*_*max*_* showed linear relationships with recovery temperature in the control and all treatments, and the small RMSE values (RMSE ≤ 0.064, data not shown) indicated that the established secondary models showed good performances. Sant’Ana et al. (2012) used the square root model to investigate the changes in growth parameters (λ and μ*_*max*_*) of *S. enterica* and *L. monocytogenes* in minimally processed lettuce as a function of temperature. The results also showed that models obtained were accurate and suitable for modeling the growth of *S. enterica* and *L. monocytogenes*. In addition, other secondary models such as hyperbolic and polynomial models have also been reported (Swinnen et al., 2004; Aguirre et al., 2013). From a practical point of view, temperature is a major environmental factor affecting bacterial growth parameters in foods. Furthermore, since the *L. monocytogenes* cells are damaged rather than being entirely killed after mild bactericidal treatments, therefore information on the growth kinetics of injured cells should be developed to help obtain reliable outputs when injured cells of foodborne pathogens are taken into account in the risk assessment.

## Conclusion

This study sought to evaluate the effects of four mild bactericidal treatments (heat, LA, BC, and nisin) on the inactivation, sublethal injury, and subsequent growth of *L. monocytogenes* cells, and the growth parameters at different recovery temperatures (20, 25, 30, and 37°C) were further determined. The obtained results demonstrated that the four bactericidal treatments induced sublethally injured cells. Percentage of sublethal injury was shown to be dependent on the type of selected bactericidal treatments. Compared to non-treated cells, mild bactericidal treatments induced significant extension of lag time of *L. monocytogenes*. Based on the correlation analysis of sublethal injury percentage on lag time, it indicated that only heat treatment showed the significant correlation. In addition, information regarding λ value of treated *L. monocytogenes* from this study was useful for determining a wide range of bacterial injury levels. Once repairing the cellular damage, the μ*_*max*_* parameter of treated *L. monocytogenes* cells was not different from non-treated cells regardless of different forms of stresses. As a whole, the results obtained in our study are valuable for helping understand the behavior of *L. monocytogenes* under various mild bactericidal treatments, and the resuscitation behavior of sublethal injured bacterial cells should be taken into account in the predictive modeling and risk assessment studies to reduce the potential food safety risks of stress injured *L. monocytogenes* cells.

## Data Availability Statement

The raw data supporting the conclusions of this article will be made available by the authors, without undue reservation.

## Author Contributions

TF was responsible for the experimental work, article writing, and data analysis. XW and QD conceived of the study and participated in its design and coordination. YW, YX, LS, XQ, YL, and HL participated in the experimental work. All authors reviewed and approved the final manuscript.

## Conflict of Interest

The authors declare that the research was conducted in the absence of any commercial or financial relationships that could be construed as a potential conflict of interest.
